# Anatomical Facial Characteristics of Teeth and Tooth Analysis

**DOI:** 10.3390/dj13010002

**Published:** 2024-12-24

**Authors:** Sybrand Gerhard de Bruin, Sundika Ishwarkumar-Govender, Pamela Pillay

**Affiliations:** 1 Department of Clinical Anatomy, School of Laboratory Medicine and Medical Sciences, University of KwaZulu-Natal, Westville Campus, Private Bag X54001, Durban 4000, South Africa; drsgdebruin@gmail.com; 2 Department of Human Anatomy and Physiology, Faculty of Health Sciences, University of Johannesburg, Doornfontein Campus, Auckland Park, P.O. Box 52, Johannesburg 2006, South Africa; sishwarkumar@uj.ac.za

**Keywords:** tooth analysis, clinical crown, 3D intra-oral scans, digital analysis

## Abstract

**Background:** This study aimed to document the angulation, inclination, and facial anatomical characteristics of teeth in a select South African population to determine if there are any population norms. Digital intra-oral scans were used, to analyze the morphology of teeth and measure the facial clinical crown. **Methods**: A quantitative observational research design with 60 3D intra-oral scans of a select South African population group was used. Morphometric analysis of 3D intra-oral scans was performed for a select South African population group, measuring the clinical crown height, width, angulation, and inclination of each tooth. **Results**: Significant differences in crown widths between male and female subjects were observed for several teeth in the maxillary and mandibular arches: males exhibited larger mean widths and larger crown dimensions than females. The South African Black group showed more sexual dimorphism compared to the South African Indian group. Clinical crown length and tooth angulation differed significantly between South African Indian and South African Black populations, while crown width and inclination remained consistent across these population groups and crown inclination between sex groups. **Conclusions**: Certain teeth exhibited notable variations between South African Indian and South African Black population groups; specifically, crown length and angulation had significant differences, whereas crown inclination and width remained consistent across these population groups.

## 1. Introduction

Human dentition is a complex and variable structure, with a sophisticated interplay of form and function, consisting of different types of dentate, each with unique anatomical features [1,2]. These unique features encapsulate a remarkable diversity that is shaped by a myriad of factors, including genetic predisposition and environmental influences [3,4]. Evolutionary processes have left an indelible imprint on the unique morphology of teeth, thus reflecting their adaptive roles in mastication and speech, as the results of a previous study have shown [5].

Each tooth’s morphology, including its crown shape, root structure, and occlusal relationships, contribute to the complexity of orthodontic bracket placement [2]. While orthodontists primarily focus on the alignment of teeth within the arch, it is crucial to recognize the significant variations in tooth anatomy among individuals [6,7].

The ‘six keys of normal occlusion’, which describe the individual and collective scheme of occlusion, showed that crown angulation (the gingival portion of the long axes of a crown being more distal than the incisal portion) and inclination (maxillary and mandibular anterior teeth having plus values and posterior teeth having minus values) played fundamental roles in the total occlusal scheme of an individual; however, these studies were based on a Caucasian sample group [8,9].

In the 1970s, researchers began exploring the application of the golden ratio in dentistry, with Lombardi first linking it to teeth proportions in 1973, although he found it ‘too strong for dental use [10]’. Levin later suggested the golden ratio for dental aesthetics, proposing that the width of the lateral incisor should be 0.618 of the central incisor, and the canine 0.618 of the lateral, focusing on the appearance from a head-on view [11]. Snow [12] proposed measuring the width of the central incisor as a percentage of the width between the canines. Additionally, an aesthetically perfect smile includes specific interproximal contact ratios: 50% contact between central incisors, 40% between central and lateral incisors, and 30% between lateral incisors and canines [13].

While some researchers consider these proportions crucial for a balanced smile, later studies have shown that adherence to golden proportions is not essential for aesthetic appeal, as many pleasing smiles do not conform to these ratios, and golden ratios are rarely observed in natural smiles [14,15,16]. Instead, the symmetrical alignment of teeth within the arch and with the facial midline is thought to be the foundational element for a balanced smile. Asymmetry in smiles is a common reason for seeking orthodontic treatment [17].

Patients are generally able to accurately assess the symmetry of their upper incisors, making this an important aspect of dental diagnostics in orthodontic and anterior tooth restoration. Clinicians should focus on achieving precise symmetry in the central incisors, as patients are more sensitive to asymmetries in these teeth, while slight asymmetries in the lateral incisors are more tolerable [18].

Other investigations have shown that there are norms for overall angulation and inclination of teeth for different population groups across the world [19,20].

This study aims to document the angulation, inclination, and facial anatomical characteristics of teeth in a select South African population.

## 2. Materials and Methods

### 2.1. Research Design

A retrospective review of digital 3D intra-oral scans of 60 patients (divided equally among South African Black males (n = 15) and females (n = 15) as well as South African Indian males (n = 15) and females (n = 15) between ages 12 and 21) was used. The data were obtained from participating private dental and orthodontic clinics within South Africa, as most public departments do not have access to 3D intra-oral scanners. The databases of the private clinics were accessed after approval from the clinics via the acceptance of the Gatekeepers letter, and patients who have had a 3D intra-oral scan performed and fall within the selection criteria were identified from the records.

This study utilized a quantitative observational research design, which incorporated methodologies from previous studies [8,21,22]. This study was performed by a single observer on 3 separate occasions.

#### 2.1.1. Clinical Crown Length and Width

Rhinocerus 3D software version 7 was used to orientate the model in the 3D space.

**Figure 1:**
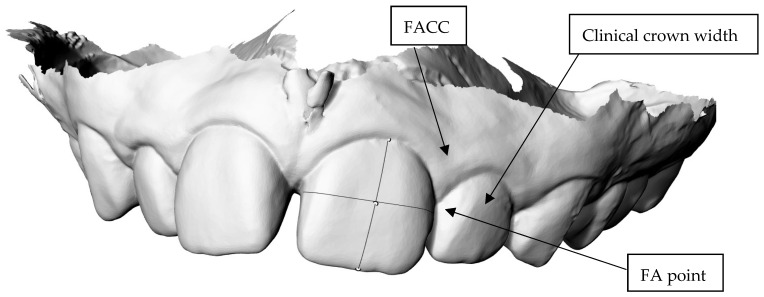
The mesiodistal line (clinical crown width), passing through the FA point on the FACC.

Measurements of the teeth were performed as follows:

- The Facial Axis Clinical Crown (FACC) was traced in an apical coronal position according to Andrews’ criteria [8]. The PolyLineOnMesh command was used starting at the most apical portion near the gingiva and moving coronally (Figure 1).
- The Facial Axis (FA) is the midpoint of the FACC.
- The curve representing the mesiodistal width of the tooth was traced through the FA point. The command Divid/Curve/Number of Segments/2 was used to divide the FACC line (Figure 1).
- PolyLineOnMesh was used with the Object Snap Function Mid and Perpendicular activated to allow for the mesial distal line to be drawn snapping to the midpoint of the FACC and perpendicular to the FACC (Figure 1).

#### 2.1.2. Clinical Crown Angulation and Inclination

The technique for assessing crown angulation and inclination was derived from [8].

The occlusal plane was defined as the common plane established by the incisal and occlusal surfaces of the teeth [23].

**Figure 2:**
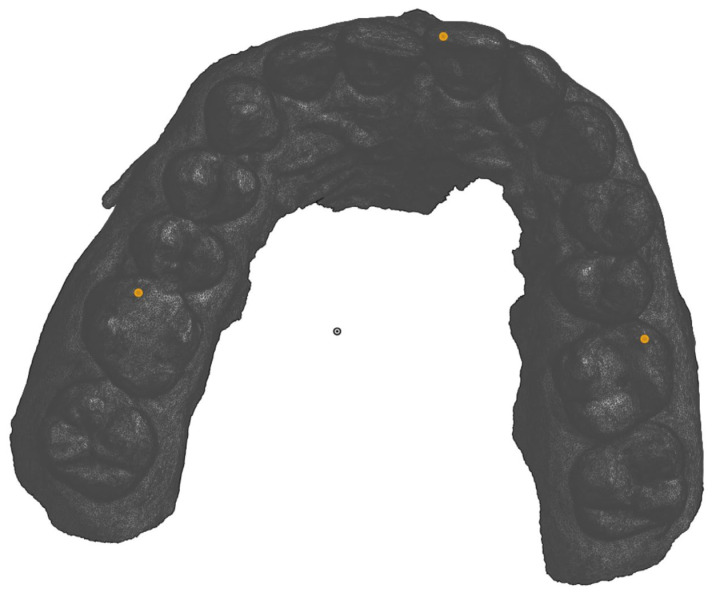
Occlusal view of the 3 points selected to form the occlusal plane.

The ‘Plane’ option in Onshape was selected and the following three points marked to construct the occlusal plane on the maxillary model (Figure 2):

- The most prominent point on the mesial buccal cusp of the first left molar.
- The most prominent point on the mesial buccal cusp of the first right molar.
- The most prominent point between the incisal tips of the central incisors.
- The same was performed for the mandibular models.

#### 2.1.3. Crown Angulation

The crown angulation is the tip of the long axis of the crown and not the long axis of the entire tooth. The long axis for all teeth except molars is the mid-developmental ridge which signifies the most prominent and centermost vertical part of the labial or facial surface of the crown. The molar crowns were identified by the buccal groove [24]. The degree of angulation is the angle between the long axis of the crown and a 90-degree line from the occlusal plane [8].

**Figure 3:**
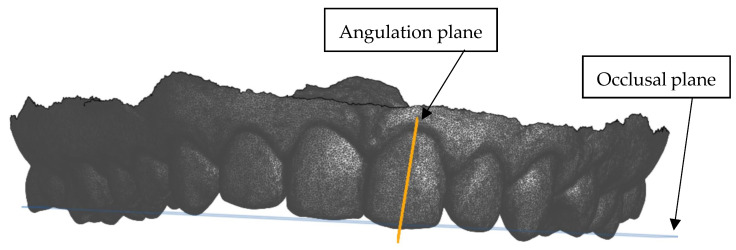
Anterior view showing the construction of the angulation plane perpendicular to the occlusal plane.

The ‘Plane’ option was selected in Onshape and the FACC was plotted, plus an additional point selected to bisect the tooth and construct the angulation plane perpendicular to the occlusal plane (Figure 3). The angulation plane and the occlusal plane were selected and the ‘Show measure details’ function used to determine the degrees of the tooth angulation perpendicular to the occlusal plane. This measurement was recorded on the Excel 365 version 2411 template for both the maxillary and mandibular teeth.

#### 2.1.4. Crown Inclination

**Figure 4:**
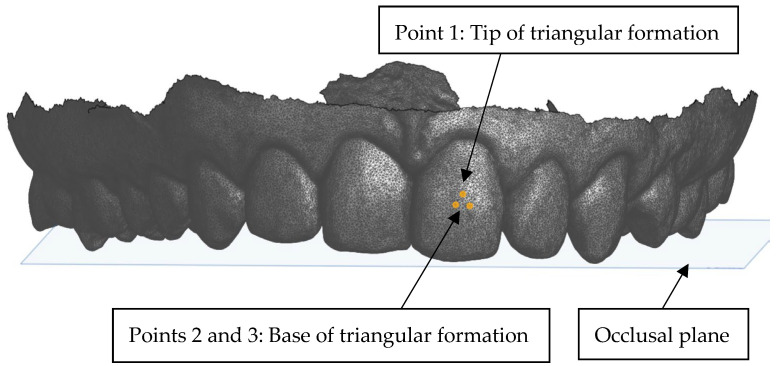
Anterior view of the three points selected to form the inclination plane.

The crown inclination is the labiolingual or buccolingual inclination of the long axis of the crown and not the entire tooth. The angle is formed by a line tangent to the middle of the labial or buccal long axis of the crown and a 90-degree line from the occlusal surface [8]. The ‘Plane’ option was selected in Onshape and three points were selected to construct the inclination plane perpendicular to the occlusal plane. The three points closest to the FA point on the facial aspect of the crown were plotted in a triangular formation (Figure 4).

**Figure 5:**
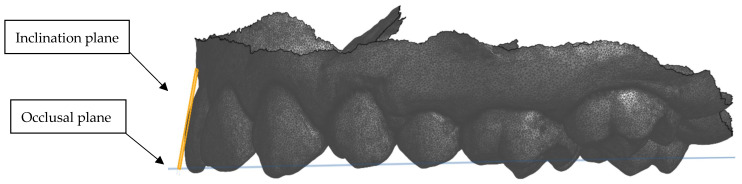
Lateral view showing the construction of the inclination plane perpendicular to the occlusal plane.

The inclination plane and the occlusal plane were selected and the ‘Show measure details’ function used to determine the degrees of the tooth inclination perpendicular to the occlusal plane (Figure 5). This measurement was recorded on the Excel template for both the maxillary and mandibular teeth.

### 2.2. Ethics Approval

The study protocol was approved by the BREC Committee, University of KwaZulu Natal, South Africa. Ethics Reference No.: BREC/00005959/2023.

### 2.3. Selection Criteria

The patients were subject to the following inclusion criteria:

- Eruption of all permanent teeth up to the first molars.
- Presence of complete permanent dentition except for the second and third molars.
- Minimal crowding to allow for accurate measurements.
- Age between 12 and 21 years at the date of the scan.

The exclusion criteria consisted of the following:

- Any teeth showing signs of wear, surface defects, alterations, or passive or active eruption.
- Teeth with restorations that affect the facial anatomy of the teeth.
- Severe rotations preventing analysis of the facial aspect of the teeth.

### 2.4. Statistical Analysis

Statistical analysis involved aggregating individual Excel files into a single CSV using Python 3.12, then calculating means taken from 3 measurements for each tooth on separate occasions, standard deviations, *t*-tests, and *p*-values for crown length, width, angulation, and inclination. Data were organized by tooth and group for comparison of sex and population differences. An independent sample *t*-test assessed morphometric differences and sexual dimorphism within and between South African Black and South African Indian populations, with significance set at *p* ≤ 0.05.

## 3. Results

**Figure 6:**
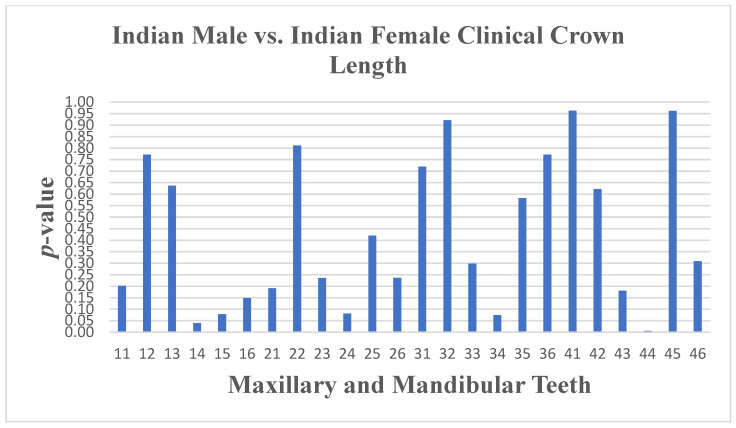
A statistical comparison analysis of clinical crown length between Indian female and Indian male subjects.

**Figure 7:**
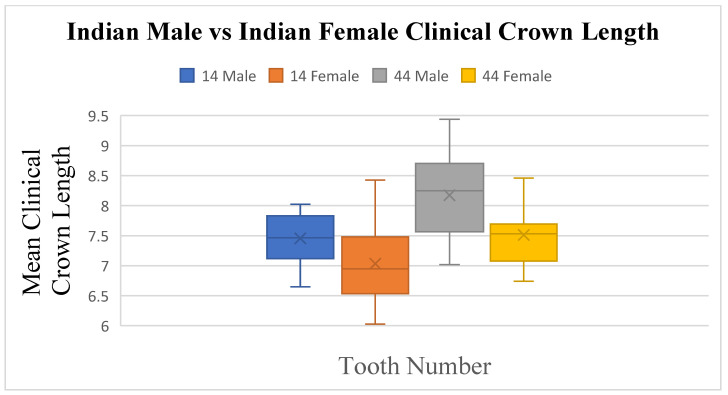
A statistical comparison analysis of the mean clinical crown length between Indian female and Indian male subjects.

An independent *t*-test was performed on both samples to monitor for any differences in tooth measurements between sexes. No real sexual dimorphism was found in the Indian population group except for the length (Figure 6 and Figure 7) of tooth 14 (*p* ≤ 0.04) and of tooth 44 (*p* ≤ 0.01), as well as the angulation of tooth 24 (*p* ≤ 0.042) and tooth 45 (*p* ≤ 0.05).

**Figure 8:**
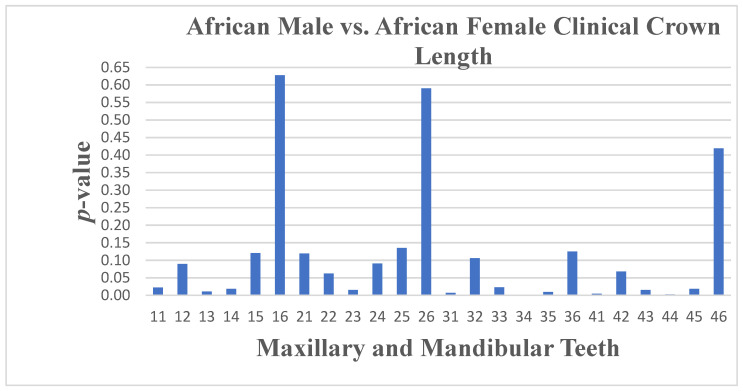
A statistical comparison analysis of clinical crown length between African female and African male subjects.

**Figure 9:**
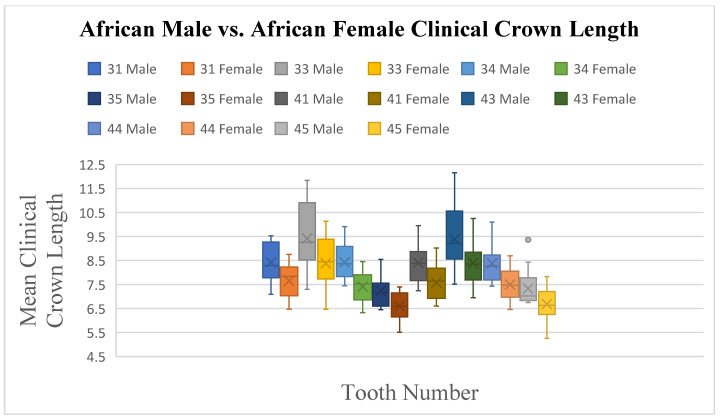
A statistical comparison analysis of the mean clinical crown length between African female and African male subjects.

There is a notable difference in the South African Black population group with regards to sexual dimorphism, with statistically significant *p*-values for teeth 11, 13, 14, and 23 of below 0.05, indicating differences in crown length (Figure 8) between South African Black males and South African Black females, with tooth 13 (*p* ≤ 0.011) showing a significant difference with a higher mean crown length in males (9.35 mm) compared to females (8.47 mm). The mandible teeth 31, 33, 34, 35, 41, 43, 44, and 45 had *p*-values below 0.05 and, specifically, tooth 33 has a *p*-value of 0.023, indicating a significant difference, with males having a higher mean crown length (9.42 mm) compared to females (8.38 mm) (Figure 9).

**Figure 10:**
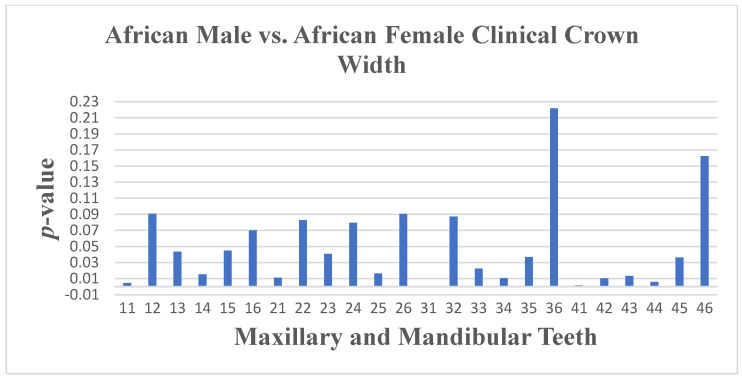
A statistical comparison analysis of clinical crown width between African female and African male subjects.

**Figure 11:**
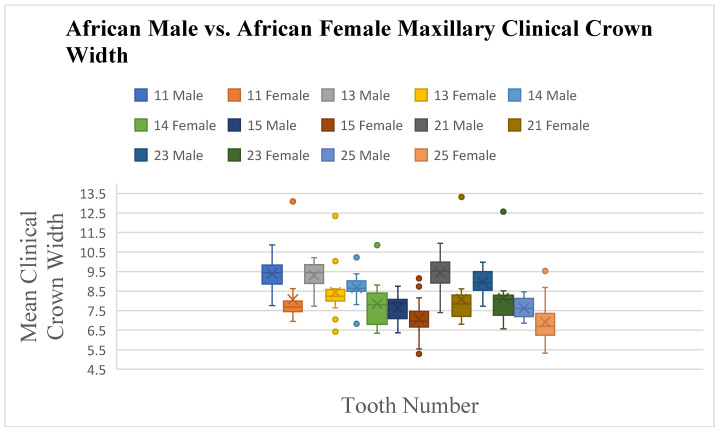
A statistical comparison analysis of the mean maxillary clinical crown width between African female and African male subjects.

**Figure 12:**
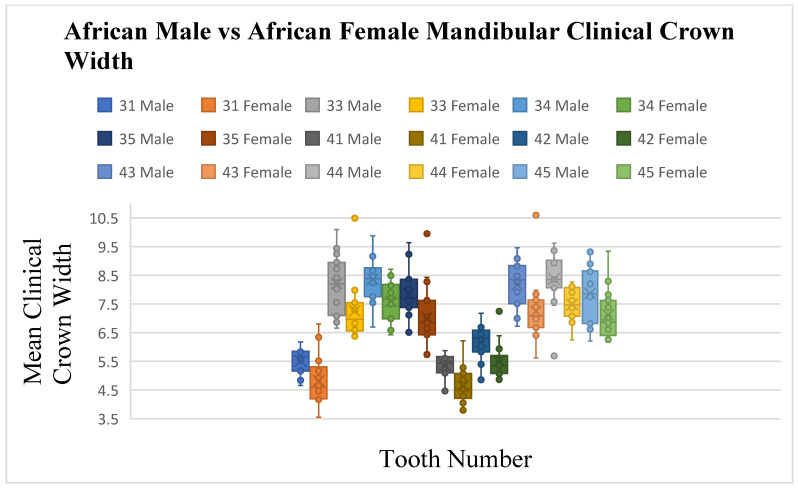
A statistical comparison analysis of the mean mandibular clinical crown width between African female and African male subjects.

Significant differences in crown width were observed between South African Black male and female subjects, with seven maxillary and nine mandibular teeth showing larger mean widths in males (*p* ≤ 0.05) (Figure 10, Figure 11 and Figure 12). Angulation differences were noted for teeth 33 and 34 (*p* ≤ 0.05), while no significant differences in crown inclination were found between South African Black males and females

**Figure 13:**
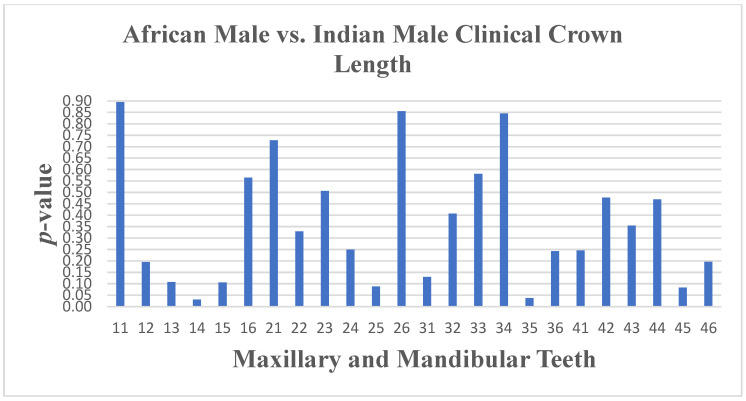
A statistical comparison analysis of clinical crown length between African male and Indian male subjects.

**Figure 14:**
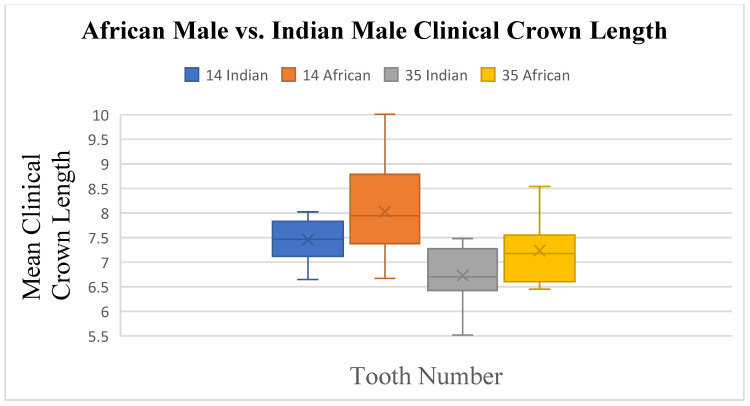
A statistical comparison analysis of the mean clinical crown length between African male and Indian male subjects.

The crown length had differences on tooth 14 (*p* ≤ 0.031) and tooth 35 (*p* ≤ 0.038). The crown tooth angulation showed a difference on tooth 33 (*p* ≤ 0.008), and the crown inclination had a difference on tooth 26 (*p* ≤ 0.009) only. Small differences were noted for South African Indian males and South African Black males in crown length (Figure 13 and Figure 14), angulation, and inclination, with no difference in width.

**Figure 15:**
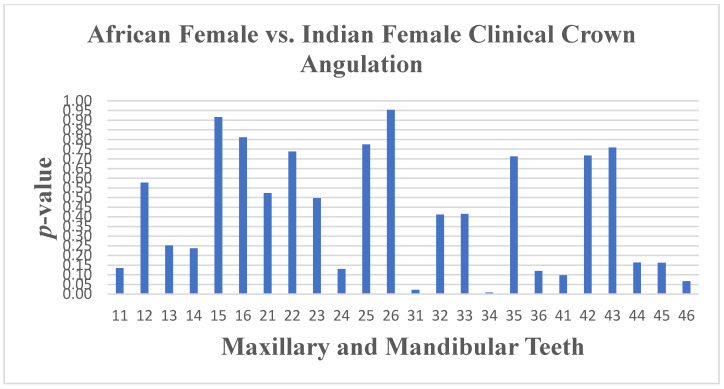
A statistical comparison analysis of clinical crown angulation between African female and Indian female subjects.

**Figure 16:**
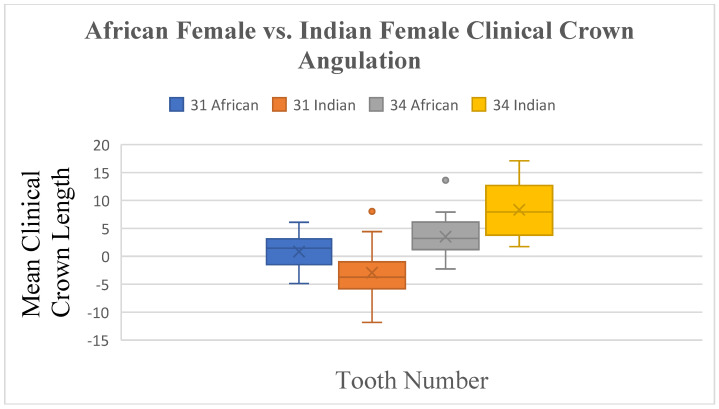
A statistical comparison analysis of the mean clinical crown angulation between African female and Indian female subjects.

Crown width and inclination were similar between South African Black and South African Indian females. However, differences were found in crown angulation for teeth 31 (*p* ≤ 0.023) and 34 (*p* ≤ 0.008), and crown length varied significantly for tooth 34 (*p* ≤ 0.015) (Figure 15 and Figure 16).

Marked differences were found between crown lengths and widths of teeth between males and females, with eleven teeth having statistically significant differences in crown length and nine teeth having significant differences in crown width. There was no difference found in the inclination between males and females, with some difference in the angulation on tooth 24 (*p* ≤ 0.046) and tooth 42 (*p* ≤ 0.046) (Table 1).

**Figure 17:**
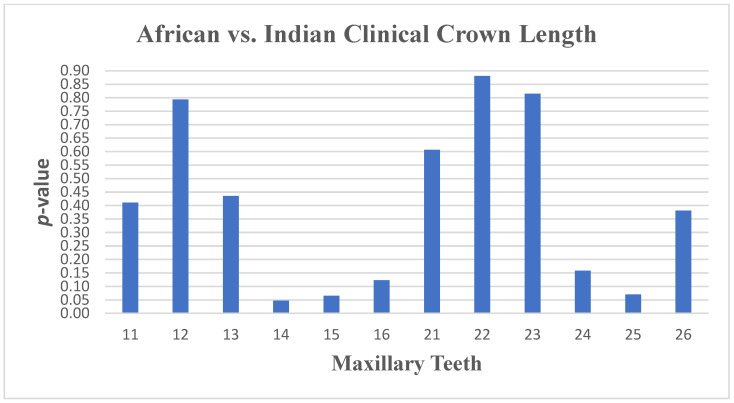
A statistical comparison analysis of maxillary clinical crown length between African and Indian samples.

**Figure 18:**
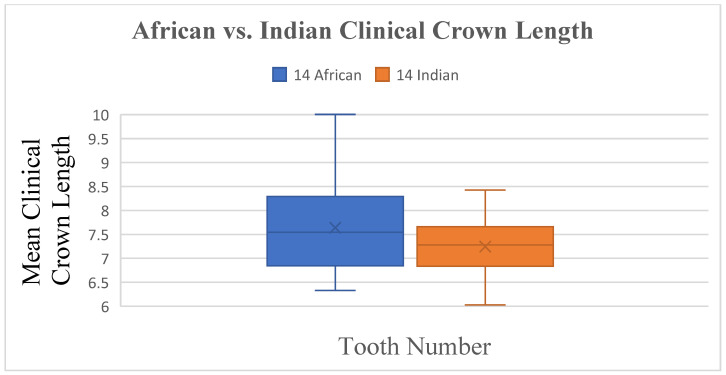
A statistical comparison analysis of the mean maxillary clinical crown length between African and Indian samples.

There were no significant differences found in the width and inclination measurements between South African Indian and South African Black populations, with only small differences in the length (Figure 17 and Figure 18) of tooth 14 (*p* ≤ 0.047) and the angulation of tooth 31 (*p* ≤ 0.007) and tooth 33 (*p* ≤ 0.015).

## 4. Discussion

The straight-wire technique was developed to minimize the need for additional bends during the treatment process [25]. The clinical outcomes are influenced by many factors of the tooth (crown and root morphology) and the design of the bracket [26]. The position of the bracket in the 3D space is still the most important aspect and is greatly influenced by the base of the bracket’s fit against the tooth surface [27]. It was recognized that a better fit of the bracket base against the tooth crown surface would facilitate more accurate bracket positioning; however, population differences in tooth position, morphology, size, and width were not taken into account, as the results of a previous study showed [9].

The effectiveness of this technique is influenced by factors such as crown and root morphology, as well as the design and fit of the bracket. Despite the recognition that a better fit of the bracket base against the tooth surface would improve bracket positioning, previous studies have shown that racial and ethnic differences in tooth position, morphology, size, and width have not always been taken into account. This oversight is important, as such variations could influence the outcome of orthodontic treatment.

This study analyzed population and sex differences in the crown height, width, angulation, and inclination of teeth between South African Indian and South African Black populations. The age range was selected to ensure eruption of all the permanent teeth had occurred and to reduce influence of tooth wear affecting measurements. The measurements were performed on digital 3D intra-oral scans, which allowed for improved direct tooth analysis and remeasurement for accuracy.

An independent two-sample *t*-test which was conducted to determine any population or sexual dimorphism differences between South African Indian and South African Black groups revealed no significant differences in crown width and crown inclination measurements. However, some specific teeth showed significant differences in crown length and angulation. The crown length for tooth 14 exhibited a significant difference, with a *p*-value of 0.047, and the crown angulation for tooth 31 and tooth 33 showed significant differences, with *p*-values of 0.007 and 0.015, respectively. These results suggest that while most measurements do not differ significantly between these ethnic groups, certain teeth exhibit notable variations.

These differences in crown morphology may have clinical implications, especially for orthodontists planning treatments, as these variations can impact bracket placement and the mechanical forces applied during treatment. Orthodontists must consider these variations to avoid inaccuracies in bracket positioning, which could lead to less predictable treatment outcomes or require adjustments during the treatment process.

Significant differences in dental measurements between male and female subjects were observed in both South African Indian and South African Black populations. Notable differences were found in crown length for eleven teeth, crown width for nine teeth, and crown angulation for teeth 24 and 42 (*p* = 0.046). Crown inclination, however, showed no significant gender differences, aligning with previous findings of positive inclination in incisors for the Marathe population, but differing in molar inclination compared to earlier studies which reported negative inclination [19].

These differences suggest that male and female patients may require different treatment approaches, particularly in populations where sexual dimorphism in dental morphology is more pronounced. In clinical practice, this could mean the need for gender-specific bracket and wire selection to achieve optimal alignment and aesthetics.

Palone et al. (2020) showed marked differences between and within the population groups of Italian and Mozambican populations regarding crown height and crown width, with the measurements of their study showing 1–2 mm differences when comparing similar tooth crown width and crown length with a South African Indian and African population group [22].

In the Indian population, minimal sexual dimorphism was observed. The significant differences noted were limited to the crown length for tooth 14, with a *p*-value of 0.04, and tooth 44, with a *p*-value of 0.01. Additionally, crown angulation showed significant differences for tooth 24 and tooth 45, with *p*-values of 0.042 and 0.05, respectively. These findings suggest that sexual dimorphism in dental measurements within the Indian group is relatively limited.

In the South African population, significant sexual dimorphism was observed in dental measurements. Differences in crown width and length were significant across both maxillary and mandibular arches, with males showing larger clinical crown width and length. Population variations in tooth width have been noted by previous researchers, and variations in tooth width are likely to be population-specific [28,29,30,31].

When comparing South African Indian and South African Black males, significant differences were found in certain dental measurements. Specifically, crown length showed significant differences for two teeth, crown angulation for one tooth, and crown inclination for one tooth. However, there was no significant difference in crown width between South African Indian and South African Black males.

In the comparison between South African Indian and South African Black females, significant differences were observed in crown length for one tooth and crown angulation for two teeth. No significant differences were noted in crown width or crown inclination between South African Indian and South African Black females.

These findings indicate that while some dental measurements differ significantly between South African Indian and South African Black males and females, others, such as crown width and inclination, remain consistent across these population groups.

This suggests that dental treatment protocols may need to be adjusted for specific ethnic groups. Adapting treatment to these variations can improve clinical outcomes and enhance the accuracy of forensic identification, ultimately improving the quality of care in both settings.

## 5. Limitations

This study was limited to South African Indian and South African Black population groups, as these were the predominant demographics seen in the orthodontic clinic’s records that were utilized. A limitation was noted during crown length and width measurements when making use of the polyline on mesh function: surface irregularities can have an effect on the outcome of the measurements due to increased deposition of enamel or buccal pits on the lower molar teeth.

## 6. Conclusions

While most dental measurements do not differ significantly between South African Indian and South African Black population groups, certain teeth exhibited notable variations. The findings of this study indicate that sex plays a role in the variation in certain dental measurements, with notable differences in crown length and width as well as angulation, and that a one-size-fits-all bracket approach in orthodontics may not be ideal.

Future developments in the straight-wire technique and other orthodontic practices must consider these anatomical and demographic differences to enhance treatment precision and effectiveness. This includes integrating digital technologies for better diagnostic and treatment planning, as well as developing guidelines that account for population and sex variations in dental morphology. By acknowledging and addressing these differences, orthodontic treatments can be more effectively tailored to individual patients, improving overall outcomes and patient satisfaction.

## Figures and Tables

**Table 1:** A statistical comparison analysis of clinical crown length and clinical crown width between male (African and Indian) and female (African and Indian) samples.

Arch	Tooth	Length, *p*-Value	Width, *p*-Value
**Maxilla**	11	0.009 *	0.048 *
	12	0.258	0.047 *
	13	0.044 *	0.043 *
	14	0.002 *	0.105
	15	0.022 *	0.168
	16	0.148	0.057
	21	0.041 *	0.018 *
	22	0.239	0.045 *
	23	0.010 *	0.043 *
	24	0.015 *	0.095
	25	0.090	0.063
	26	0.196	0.064
**Mandible**	31	0.148	0.047 *
	32	0.257	0.083
	33	0.020 *	0.049 *
	34	0.000 *	0.065
	35	0.132	0.072
	36	0.502	0.107
	41	0.059	0.053
	42	0.124	0.068
	43	0.007 *	0.026 *
	44	0.000 *	0.079
	45	0.059	0.126
	46	0.839	0.059

Analysis was performed using a two-sample *t*-test, and measurements are in mm. * *p* ≤ 0.05.

## Data Availability

The dataset analyzed during this article is available from the corresponding author on request.

## References

[1] Morris A.L., Tadi P. (2023). Anatomy, Head and Neck, Teeth.

[2] Robert J. (2023). Dental Anatomy Understanding the Structure and Function of Teeth. J. Interdiscipl. Med. Dent. Sci..

[3] Townsend G., Harris E.F., Lesot H., Clauss F., Brook A. (2009). Morphogenetic fields within the human dentition: A new, clinically relevant synthesis of an old concept. Arch. Oral. Bio..

[4] Khan M.I., Ahmed N., Kumar Neela P., Unnisa N., Khan I. (2022). The Human Genetics of Dental Anomalies. Glob. Med. Genet..

[5] Scott G.R., Turner C.G. (1997). The Anthropology of Modern Human Teeth: Dental Morphology and Its Variation in Recent Human Populations.

[6] Otuyemi O.D., Noar J.H. (1996). A comparison of crown size dimensions of the permanent teeth in a Nigerian and a British population. Eur. J. Orthod..

[7] Salih A., Al-Qudah Y., Al-Azzeh H., Al-Khalaf M., Abu-Halaweh S., Al-Hamad M. (2020). Vestibular Height of Teeth in Different Racial and Ethnic Groups For the Use of Fixed Orthodontic Treatment: A Systematic Review. J. Clin. Exp. Dent..

[8] Andrews L.F. (1972). The six keys to normal occlusion. Am. J. Orthod..

[9] Andrews L.F. (1976). The straight-wire appliance. Explained and compared. J. Clin. Orthod..

[10] Lombardi R.E. (1973). The Principles of Visual Perception and Their Clinical Application to Denture Esthetics. J. Prosthet. Dent..

[11] Levin E.I. (1978). Dental Esthetics and the Golden Proportion. J. Prosthet. Dent..

[12] Snow S.R. (1999). Esthetic Smile Analysis of Maxillary Anterior Tooth Width: The Golden Percentage. J. Esthet. Dent..

[13] Rayyan M.R. (2019). Effect of the Interproximal Contact Level on the Perception of Smile Esthetics. Dent. Med. Probl..

[14] Magne P., Salem P., Magne M. (2018). Influence of Symmetry and Balance on Visual Perception of a White Female Smile. J. Prosthet. Dent..

[15] Londono J., Ghasemi S., Lawand G., Dashti M. (2023). Evaluation of the Golden Proportion in the Natural Dentition: A Systematic Review and Meta-Analysis. J. Prosthet. Dent..

[16] Ahmed N., Halim M.S., Khalid S., Ghani Z.A., Jamayet N.B. (2022). Evaluation of Golden Percentage in Natural Maxillary Anterior Teeth Width: A Systematic Review. J. Prosthet. Dent..

[17] Sarul M., Antoszewska-Smith J., Park H.S. (2019). Self-Perception of Smile Attractiveness as a Reliable Predictor of Increased Patient Compliance with an Orthodontist. Adv. Clin. Exp. Med..

[18] Chrapla P., Paradowska-Stolarz A., Skoskiewicz-Malinowska K. (2022). Subjective and Objective Evaluation of the Symmetry of Maxillary Incisors among Residents of Southwest Poland. Symmetry.

[19] Kamble S., Deshmukh S., Durkar S., Rahalkar J. (2017). Establishment of norms for crown angulation and inclination among Maratha population: Cross-Sectional Study. Int. Dent. J. Stud. Res..

[20] Halim H., Halim I.A. (2020). The study of tooth angulation and inclination for bracket design of Deutromalay race: A comparison study to white race. J. World. Fed. Orthod..

[21] Dindaroğlu F., Duran G.S., Aras I. (2016). Three-dimensional evaluation of morphologic tooth symmetry in various malocclusions. Am. J. Orthod. Dentofacial. Orthop..

[22] Palone M., Spedicato G.A., Lombardo L. (2020). Analysis of tooth anatomy in adults with ideal occlusion: A preliminary study. Am. J. Orthod. Dentofacial. Orthop..

[23] (2005). The glossary of prosthodontic terms. J. Prosthet. Dent..

[24] Doodamani G.M., Khala A.S., Manohar M., Umashankar (2011). Assessment of crown angulations, crown inclinations, and tooth size discrepancies in a South Indian population. Contemp. Clin. Dent..

[25] Miethke R.R., Melsen B. (1999). Effect of variation in tooth morphology and bracket position on first and third order correction with preadjusted appliances. Am. J. Orthod. Dentofacial. Orthop..

[26] Mundhada V.V., Jadhav V.V., Reche A. (2023). A Review on Orthodontic Brackets and Their Application in Clinical Orthodontics. Cureus..

[27] McLaughlin R.P., Bennett J.C. (2015). Evolution of treatment mechanics and contemporary appliance design in orthodontics: A 40-year perspective. Am. J. Orthod. Dentofacial. Orthop..

[28] Arya B.S., Savara B.S., Thomas D., Clarkson Q. (1974). Relation of sex and occlusion to mesiodistal tooth size. Am. J. Orthod..

[29] Murshid Z., Hashim H.A. (1993). Mesiodistal tooth width in Saudi population. A preliminary report. Saudi. Dent. J..

[30] Singh S.P., Goyal A. (2006). Mesiodistal crown dimension of the permanent dentition in North Indian children. J. Indian Soc. Pedod. Prev. Dent..

[31] Bugaighis I., Elorfi S. (2013). An odontometric study of tooth size in normal, crowded and spaced dentitions. J. Orthod. Sci..

